# Editorial: Reviews in pharmacology of ion channels and channelopathies

**DOI:** 10.3389/fphar.2024.1449967

**Published:** 2024-08-20

**Authors:** Jinwei Zhang, Jean-Marc Sabatier, Mohamed Chahine, Domenico Tricarico

**Affiliations:** ^1^ State Key Laboratory of Chemical Biology, Research Center of Chemical Kinomics, Shanghai Institute of Organic Chemistry, Chinese Academy of Sciences, Shanghai, China; ^2^ Xiamen Cardiovascular Hospital Xiamen University, School of Medicine, Xiamen University, Xiamen, China; ^3^ Institute of Biomedical and Clinical Sciences, Medical School, Faculty of Health and Life Sciences, University of Exeter, Hatherly Laboratories, Streatham Campus, Exeter, United Kingdom; ^4^ Institut de Neurophysiopathologie (INP), Aix-Marseille Université, CNRS UMR 7051, Marseille, France; ^5^ Department of Medicine, Faculty of Medicine, CERVO Brain Research Centre, Institut Universitaire en Santé Mentale de Québec, Université Laval, Quebec City, QC, Canada; ^6^ Department of Pharmacy-Pharmaceutical Science, University of Bari Aldo Moro, Bari, Italy

**Keywords:** ion channels, channelopathies, reviews, pharmacology, ion transporter

In the realm of pharmacology, the study of ion channels and channelopathies continues to be a fascinating and critical area, providing profound insights into cellular mechanisms and therapeutic potentials. The year 2023 has brought significant advancements, as demonstrated by five seminal review publications in this field.

The TRPV1 channel, known as the heat and capsaicin receptor, is extensively expressed in the nerve terminals of dorsal root ganglia (DRGs) and trigeminal ganglia, which innervate the body and face, respectively. It is also present in other tissues and organs, including the central nervous system. TRPV1 is a versatile receptor that detects harmful heat, pain, and a variety of internal and external ligands (Caterina et al., 1997). Amaya-Rodriguez et al. offer a thorough examination of the TRPV1 channel, its roles in physiology and pathology, and the use of *in silico* tools for drug discovery targeting this channel. They discuss the widespread expression of TRPV1 in nerve terminals and various tissues, its involvement in detecting harmful stimuli, and its links to neuroinflammation, cancer, and pathological pain. The challenges in discovering specific and efficient drugs targeting TRPV1 and the potential of *in silico* tools for molecular modelling and drug discovery are also highlighted.

Calcium cations (Ca^2+^) are vital for cellular functions. Calcium overload, marked by excessive intracellular Ca^2+^, causes irreversible cell death. Thus, calcium overload-based ion interference therapy could potentially overcome resistance to conventional tumor treatments and holds clinical promise (Bai et al., 2022). Li et al. review the potential of calcium overload-based ion interference therapy in tumor treatment. They explore the strategies used, the resulting tumor cell death, and the prospects for clinical application. The review discusses the significance of calcium overload in disrupting tumor cell function, the potential synergistic effects with immunotherapy, and the challenges and risks, such as achieving a balance between cell death and proliferation and managing systemic toxic side effects.

Large Conductance Voltage- and Calcium-activated K^+^ (BK) channels, which are transmembrane proteins activated by voltage and calcium, regulate cell excitability and are found in both excitable and non-excitable cells. They influence vascular tone, neuronal activity, neurotransmitter release, and muscle contraction (Ancatén-González et al., 2023). BK channel dysfunction can result in hypertension, hearing disorders, epilepsy, and ataxia. Echeverría et al. provide a detailed overview of BK channels in various physiological systems and their roles in different diseases. They discuss the structure and activation of BK channels, their function in regulating cellular excitability, and their influence in the nervous system, endocrine system, and other physiological systems. The review also addresses the implications of abnormal BK channel function in diseases like arterial hypertension, hearing disorders, epilepsy, and ataxia, and highlights potential therapeutic interventions for BK channelopathies.

Astrocytes are crucial for central nervous system (CNS) homeostasis, managing ionic and pH balance, neurotransmission, cerebral blood flow, and metabolism. They undergo reactive astrogliosis in response to CNS insults, inflammation, and diseases (Bélanger and Magistretti, 2022). Rahman et al. examine the relationship between reactive astrogliosis, ion transporter cascades, and cerebrovascular diseases. They emphasize the critical role of astrocytes in brain health and cerebral blood flow regulation. The review identifies potential therapeutic targets and strategies, including specific inhibitors and antibodies, to mitigate reactive astrogliosis and address cerebrovascular diseases, particularly in disorders like post-stroke dementia (PSD) and vascular contributions to cognitive impairment and dementia (VCID).

Voltage-gated sodium channel (Na_V_1.4), a voltage-gated sodium channel primarily found in skeletal muscle cells, is essential for action potentials and muscle contraction. Mutations in Na_V_1.4 can result in muscle disorders (Maggi et al., 2020). Zou et al. review the pharmacological properties of NaV1.4, discussing its structure, role in muscle function, and association with muscle disorders. They also explore the potential of drugs and toxins that interact with Na_V_1.4 as research tools or clinical agents for treating Na_V_1.4 channelopathies.

The 2023 edition of Reviews in Pharmacology of Ion Channels and Channelopathies highlights the cutting-edge research and innovation in this field. The Research Topic of reviews bridges basic science and clinical applications, offering diverse insights into the mechanisms of channel dysfunction and potential therapeutic strategies. This interdisciplinary approach underscores the importance of collaboration among researchers, clinicians, and industry partners in advancing knowledge and improving patient outcomes.

As we look forward to the future, ion channels and channelopathies will undoubtedly play a crucial role in shaping the landscape of medicine. The collective efforts of the scientific community, embodied in this year’s edition, reflect a commitment to inquiry, collaboration, and innovation. Let us continue to push the boundaries of knowledge and explore new pathways towards a healthier future.

Together, we embark on this journey, guided by the exploration of ion channels and channelopathies, unlocking the potential of pharmacology to transform lives.

## References

[B1] Ancatén-González C. Segura I. Alvarado-Sánchez R. Chávez A. E. Latorre R. (2023). Ca2+- and voltage-activated K+ (BK) channels in the nervous system: one gene, a myriad of physiological functions. Int. J. Mol. Sci. 24, 3407. 10.3390/ijms24043407 36834817 PMC9967218

[B2] Bai S. Lan Y. Fu S. Cheng H. Lu Z. Liu G. (2022). Connecting calcium-based nanomaterials and cancer: from diagnosis to therapy. Nano-Micro Lett. 14, 145. 10.1007/s40820-022-00894-6 PMC9294135 35849180

[B3] Bélanger M. Magistretti P. J. (2022). The role of astroglia in neuroprotection. Dialogues Clin. Neurosci. 11, 281–295. 10.31887/dcns.2009.11.3/mbelanger PMC3181926 19877496

[B4] Caterina M. J. Schumacher M. A. Tominaga M. Rosen T. A. Levine J. D. Julius D. (1997). The capsaicin receptor: a heat-activated ion channel in the pain pathway. Nature 389, 816–824. 10.1038/39807 9349813

[B5] Maggi L. Brugnoni R. Canioni E. Tonin P. Saletti V. Sola P. (2020). Editorial: new insights in skeletal muscle channelopathies - a rapidly expanding field. Front. Neurology 11, 626772. 10.3389/fneur.2020.626772 PMC7774510 33391177

